# Non-surgical Root Canal Treatment of an Upper First Molar With an Unusual Morphology: A Case Report

**DOI:** 10.7759/cureus.39725

**Published:** 2023-05-30

**Authors:** Ahmed A Alzahrani, Muhnnad A Balbaid, Ahmed T Fawzy

**Affiliations:** 1 Dentistry, Umm Al-Qura University, Makkah, SAU; 2 Endodontics, Umm Al-Qura University, Makkah, SAU

**Keywords:** root anatomy, root canal treatment, two roots, rare morphology, maxillary first molar

## Abstract

This paper presents the unusual morphology of an upper right first molar with two roots, each containing a single canal, in a patient seeking emergency endodontic treatment. Clinical and radiographic examinations revealed the unusual root canal morphology of the tooth, which required further investigation using cone beam computed tomography (CBCT) imaging, which confirmed this unusual anatomical structure. It was also noted that the upper right first molar was asymmetrical to the upper left first molar, which had the normal three-root morphology. The buccal and palatal canals were instrumented using ProTaper Next Ni-Ti rotary instruments and enlarged to ISO size 30, with a taper of 0.7; irrigated with 2.5% NaOCl; filled with gutta-percha using the warm-vertical-compaction technique, with the aid of a dental operating microscope (DOM); and then confirmed via periapical radiograph. The DOM and CBCT are valuable aids that helped us to confirm the endodontic diagnosis and treatment of this unusual morphology.

## Introduction

Chemo-mechanical preparation and obturation are prerequisites for successful root canal treatment (RCT). Therefore, proper knowledge of the variation of the anatomy of the root canal system is necessary for endodontic therapy to be successful [1]. Root canals range widely in terms of morphology and anatomy. Due to the upper first molar's complex anatomy, it has the highest failure rate in clinical practice [2,3]. Several studies have investigated the morphology of the maxillary first molar and revealed that it exhibits a large variation in the number of canals [4]. The upper first molar usually has three roots and four canals [5]. Moreover, the upper first molar may have five or six root canals or a C-shaped canal configuration [6,7]. RCT requires adequate knowledge of the root canal's anatomy, as well as highly specialized materials and equipment. Cone beam computed tomography (CBCT), one of the most recent advances in dentistry, is frequently utilized to produce three-dimensional (3D) images of teeth. This is now a crucial tool for diagnosis and treatment planning [8,9], as well as the evaluation of anatomical features such as isthmus and lateral canals, resulting in more consistent and successful treatment [10]. Furthermore, the dental operating microscope (DOM) is an essential tool, as it provides better visualization, which aids the practitioner in identifying complex anatomy clinically. The patient described in this study had a maxillary right first molar with unusual root canal morphology, specifically two roots and one canal in each root, which was detected and treated with the help of CBCT and a DOM. Similar anatomical structures have been documented in only a small number of existing studies [11,12]. Our case is different from the previously published cases in addition to the unusual morphology; it was asymmetrical to the other side of the upper quadrant. The DOM and CBCT are valuable aids that helped us to confirm the endodontic diagnosis and treatment of this unusual case, as they can help in the management of these types of unusual morphological variations.

## Case presentation

**Figure 1:**
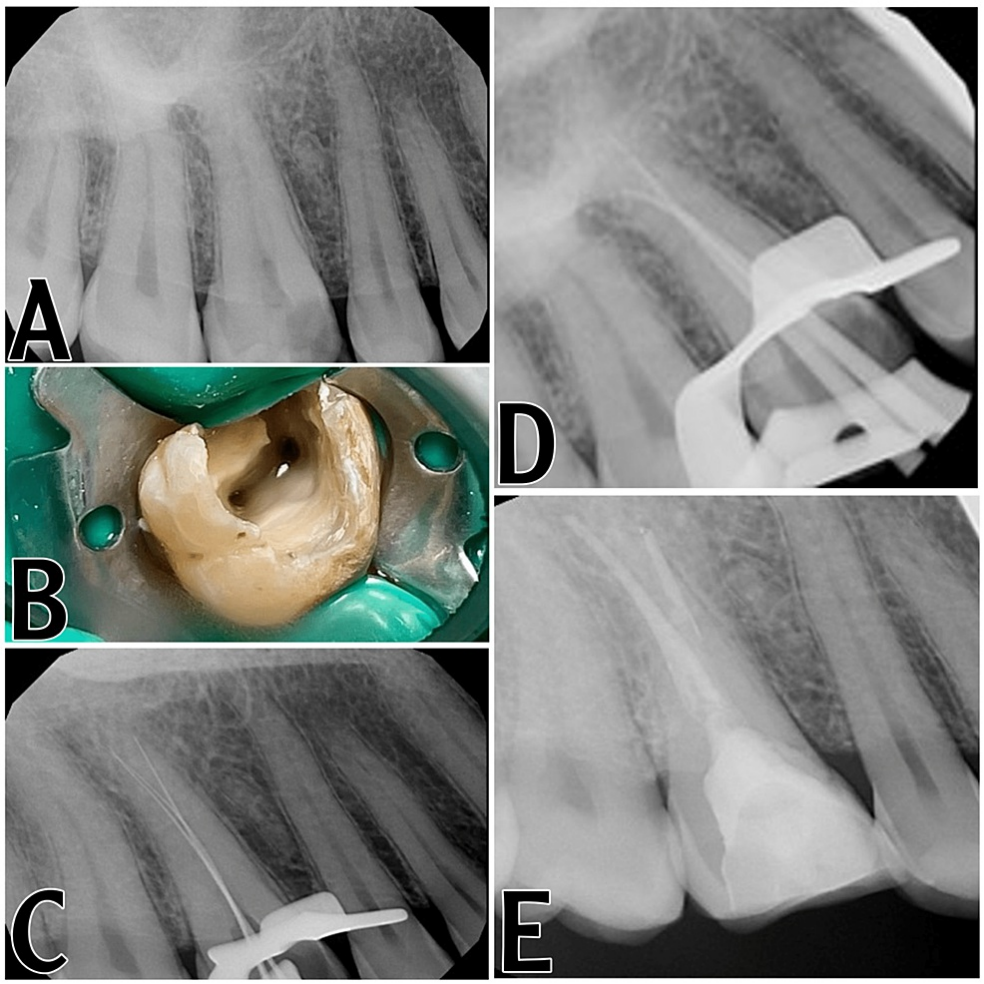
(A) A preoperative radiograph. (B) Clinical image of the identified orifices (buccal and palatal). (C) Working length determination. We added an extra file to see if we are in the same canal or if there is an apical split. (D) Master cone radiograph. (E) Final radiograph showing obturation, post, and core

A 45-year-old male patient seeking emergency treatment arrived at the outpatient clinic operated by the Faculty of Dentistry of Umm Al Qura University in Makkah, Saudi Arabia. The patient has been experiencing spontaneous pain affecting his sleep quality in the upper right quadrant for the past three days. Medically, the patient was fit and healthy. A clinical examination revealed that the maxillary right first molar had occluso-mesial decay, and the tooth had normal sensitivity to percussion and palpation. Sensitivity testing in the form of a cold test (Endo-Ice) revealed a severe lingering response; using the pain scale, it was nine out of ten. A periapical radiograph was taken by sensor size two, revealing a normal periapical area (Figure 1A). A diagnosis of symptomatic irreversible pulpits with normal periapical tissues was reached. The patient's desire was to extract the tooth because of the pain, but after explaining to the patient that the tooth was restorable, he preferred to maintain it, and the patient agreed to undergo RCT. The patient signed a consent form and was then anesthetized as buccal, palatal infiltration using 4% articaine with adrenaline at a ratio of 1:100,000; it is safe and has a greater depth of action. The tooth was isolated using a rubber dam, the caries were excavated, and the cavity was accessed with a round carbide bur size 4. There was copious bleeding and deroofing of the pulp chamber with an Endo Z tapered safe-end bur (Dentsply, Maillefer), and the root canals were negotiated with DG explorer and instrumented manually to create a glide path using size 10 stainless steel K files (Dentsply Sirona). The radicular pulp was removed using a barbed broach (Figure 1B).

**Figure 2:**
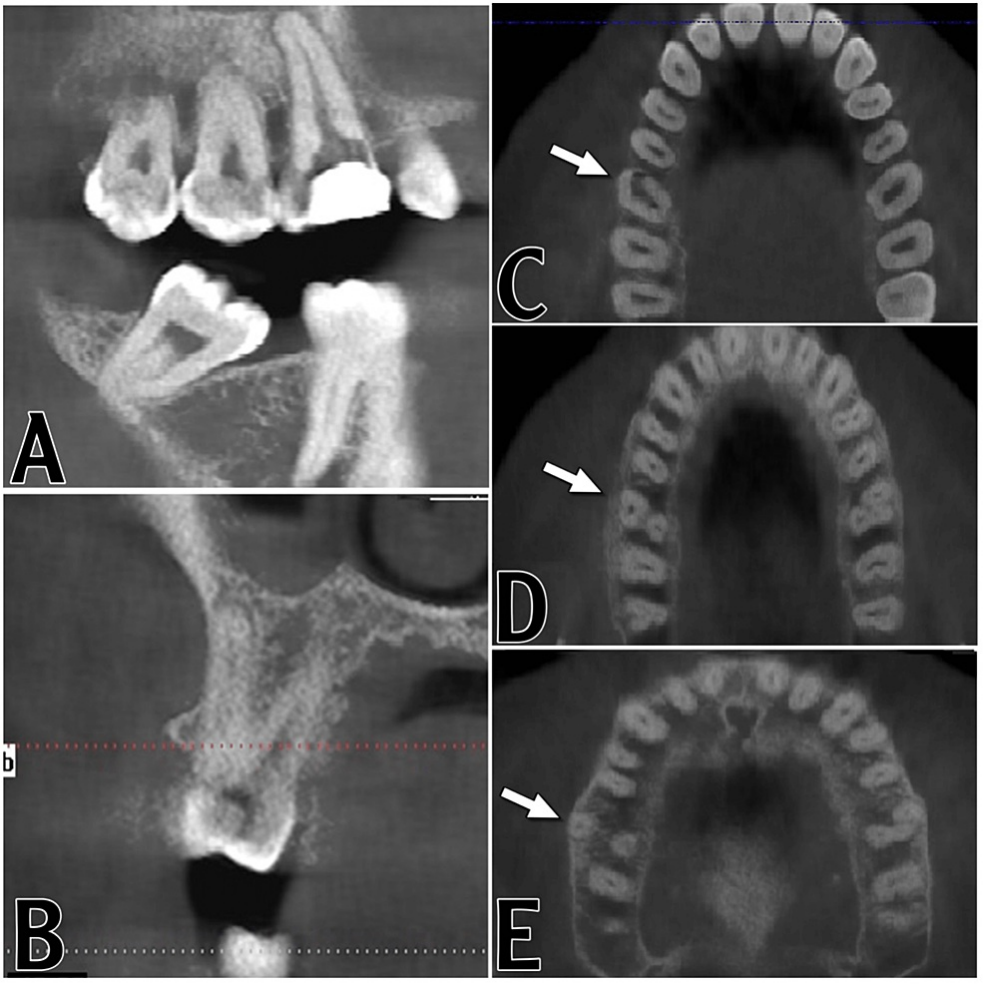
CBCT images. (A) Sagittal view of the maxillary and mandibular molars. (B) Coronal view of the maxillary right first molar. (C) Axial slice in the cervical third of the maxillary teeth. The white arrow is pointing to the unusual upper first molar. (D) Axial slice in the middle third of the maxillary teeth. The upper right first molar was asymmetrical to the upper left first molar. (E) Axial slice of the maxillary teeth in the apical third

The endodontic map shows two orifices, one on the buccal side and the other on the palatal side; 2.5% NaOCl and ethylenediaminetetraacetic acid were used to flush the canals, and the canals were dried using paper points. The tooth was then sealed with temporary filling for further investigation and CBCT scanning. The patient was then referred to the radiology department for CBCT imaging. The CBCT scan was analyzed in all three planes, specifically the sagittal, coronal, and axial (Figure 2A-2E). The CBCT confirmed the unusual morphological condition that had been noted clinically. The radiographic finding of the CBCT showing the maxillary first molar exhibited only two roots, with one canal in each root. It was also noted that the upper right first molar was asymmetrical to the upper left first molar, which had the normal three-root morphology (Figure 2D).

During the second visit, a rubber dam was put in place after the administration of anesthesia, the same type and route of administration as the first visit, and the temporary filling was removed. The working length was measured using a traditional Root ZX II apex locator display reading of 0.5 mm (Morita Corporation, Kyoto, Japan) and confirmed with a periapical radiograph (Figure 1C). The canals were instrumented using ProTaper Next Ni-Ti rotary instruments (Dentsply) to size ISO 30, with a taper of 0.7 (X3 file). The canals were then irrigated with 2.5% NaOCl, and the canals were dried using paper points. The tooth was then filled with gutta-percha using the warm-vertical-compaction technique. The tooth was then restored using a prefabricated fiber post (TiaDent) and composite core (EsCom) (Figure 1E). The patient was referred to his dentist for cuspal coverage restoration.

## Discussion

The root canal morphology of the maxillary first molar is one of the most complex root canal anatomies in human dentition [13]. According to Vertucci’s classification, the anatomy of the palatal and distobuccal roots is simple, with only one canal and a predominate type I configuration. Type I mesio-buccal roots were the most prevalent, with Type II and Type IV coming in second mesio-buccal [14]. Generally, the most frequent pattern of the maxillary permanent first molar in the literature has three roots and four canals with a high incidence of a second canal in the mesio-buccal root (MB2). As a result, most publications concerning the variability of the upper first molars focus on the MB2 canal morphology. However, the two-rooted form of the upper first molar is infrequently documented [15]. Analysis of the upper first molar's root canal morphology in the Saudi population showed two roots at a rate of 0.2% and 2.0% teeth with two canals [16].

Root fusion is thought to be caused either by cementum deposition over time or due to the inability of the Hertwig epithelial sheath to form or fuse at the furcation area [17]. Root fusion in the maxillary molars may frequently show different fusion patterns, such as partial or complete fusion of two or more roots. The fusion of two buccal roots is one of the aberrations of maxillary molars. A total of 0.4% of first maxillary molars have been reported to have this anomaly [18].

In the current case report, the RCT of a maxillary first molar with two roots and one canal in each root was asymmetrical between the right and left quadrants. According to one study, 70% to 81% of root and canal morphologies were symmetrical on the left and right sides [19]. It was, therefore, intriguing to discover that, in our case, the morphologies of the upper first molars were asymmetrical between the right and left quadrants. Clinicians must understand and treat the complete root canal system using the tools available to aid in successful RCT [20]. The clinician should also be aware of the possibility of the presence of fewer root and/or canal numbers.

In a few clinical scenarios, preoperative CBCT enables dental practitioners to investigate intricate root canal anatomy and maintain tooth structure. Because CBCT is more sensitive than conventional radiography in revealing the number of canals, its usage in endodontics has risen. Conventional radiography is inferior to CBCT for identifying and separating internal and external root resorption cavities that are in the early stages of development. Due to the underestimation of the size of periapical lesions and the ability to detect periapical lesions before they are seen on conventional radiographs, CBCT is used to detect periapical lesions associated with endodontic infection [21]. CBCT was utilized to confirm the unusual morphology and help to diagnose and compare the same tooth on the opposite side. This case report highlights the role of CBCT as a tool to reveal the 3D anatomy of teeth and unusual root morphologies.

In addition, using a DOM in routine clinical practice may further aid in the location and management of canals due to its substantially increased magnification and lightening of the field of view [22]. DOM aided in a significant increase of the operative field's eyesight, which assisted us in the obturation.

Variations in dental anatomy are found in most teeth. Knowledge of these variations, particularly regarding the location and treatment of all canals, is very important for successful endodontic treatment, because the inability to locate and properly treat the root canals may cause failures. It is the duty of dental clinicians to make the proper diagnosis and administer the appropriate care. Conducting an incorrect endodontics procedure may be considered malpractice, which may have medicolegal repercussions [23].

**Figure 3:**
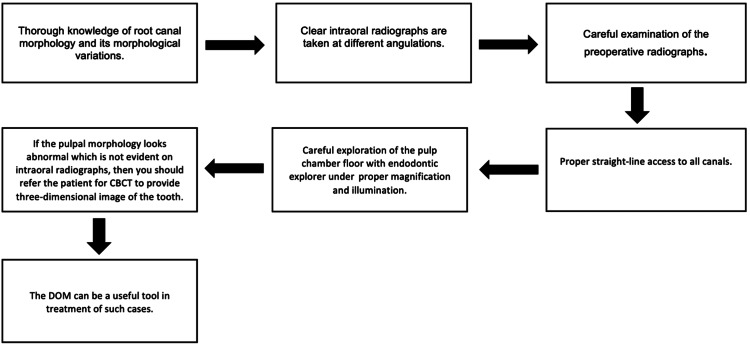
Guideline for identifying unusual morphological variations

Figure 3 gives brief tips on dealing with morphological variation through the assessment of CBCT and the use of DOM.

## Conclusions

A proper understanding of the variation in the root canal system's morphology is necessary for effective endodontic treatment. Proper diagnosis and treatment planning is mandatory prior to any endodontic treatment in order to understand and identify any morphological variations that could encounter the clinician. This would aid in successful endodontic treatment and minimize the risks of mishaps during RCT.
